# Temporal and Spatial Patterns of Sediment Microbial Communities and Driving Environment Variables in a Shallow Temperate Mountain River

**DOI:** 10.3390/microorganisms10040816

**Published:** 2022-04-14

**Authors:** Wang Tian, Huayong Zhang, Yuhao Guo, Zhongyu Wang, Tousheng Huang

**Affiliations:** Research Center for Engineering Ecology and Nonlinear Science, North China Electric Power University, Beijing 102206, China; tianwang@ncepu.edu.cn (W.T.); youhl1995@163.com (Y.G.); zhy_wang@ncepu.edu.cn (Z.W.); tous_huang@ncepu.edu.cn (T.H.)

**Keywords:** microbial community, water environment, sediment environment, temporal and spatial patterns, structure equation model, canonical correspondence analysis

## Abstract

Microbial communities in sediment play an important role in the circulation of nutrients in aquatic ecosystems. In this study, the main environmental factors and sediment microbial communities were investigated bimonthly from August 2018 to June 2020 at River Taizicheng, a shallow temperate mountain river at the core area of the 2022 Winter Olympics. Microbial community structure was analyzed using 16S rRNA genes (bacteria 16S V3 + V4 and archaea 16S V4 + V5) and high-throughput sequencing technologies. Structure equation model (SEM) and canonical correspondence analysis (CCA) were used to explore the driving environmental factors of the microbial community. Our results showed that the diversity indices of the microbial community were positively influenced by sediment nutrients but negatively affected by water nutrients. Bacteroidetes and Proteobacteria were the most dominant phyla. The best-fitted SEM model indicated that environmental variables not only affected community abundance directly, but also indirectly through influencing their diversity. *Flavobacterium*, *Arenimonas* and *Terrimonas* were the dominant genera as a result of enriched nutrients. The microbial community had high spatial–temporal autocorrelation. CCA showed that DO, WT and various forms of phosphorus were the main variables affecting the temporal and spatial patterns of the microbial community in the river. The results will be helpful in understanding the driving factors of microbial communities in temperate monsoon areas.

## 1. Introduction

Microbial communities in sediment play an important role in material circulation since they take part in the biogeochemical cycles in aquatic ecosystems. Their composition and structure are determined by the physical and chemical variables of both water and sediment [1]. In turn, microbial communities influence the environmental conditions of water and sediment, or even affect the structure and function of aquatic ecosystems [2,3]. In many lakes, the release of nitrogen and phosphorus by microbial communities is the main nutrient supply and one of the causes of water eutrophication [4]. Microbial communities are also used as biological approaches to remove toxic substances, such as soluble metals and sulfates, from water environment [5,6]. Exploring the microbial community structure and influencing factors is important in developing appropriate protection strategies in aquatic ecosystems.

Microbial communities generally have high levels of metabolic rates and efficient trophic transfer due to their small body masses. They interact with the environment through a series of biological reactions and thus are sensitively affected by a variety of environmental variables, including temperature, dissolved oxygen (DO), pH, total nitrogen (TN), total phosphorus (TP), ammonia nitrogen (NH_4_^+^-N), nitrate nitrogen (NO_3_-N), total organic carbon (TOC) and other factors of both water and sediments [7,8,9,10,11]. Some photosynthetic bacteria fix carbon dioxide through photosynthesis, while some nitrifying bacteria fix carbon dioxide by chemoautotrophic processes [12]. Methanogens can consume carbon dioxide and produce methane under anaerobic conditions [13]. Microbial taxa can also conduct biochemical reactions such as nitrogen fixation, ammonification, and nitrification and denitrification [14]. Nitrogen and phosphorus are the original materials of these reactions. Other variables such as temperature will influence the efficiency of these processes. Hou et al. [15] studied sediment microbial community distribution and found a negative relationship between abundance and sediment organic matter in Lake Guozheng and Lake Tuanhu. Zhao et al. [16] and Liu et al. [17] showed that sediment microbial community abundance was influenced by pH, NH_4_^+^-N, NO_3_^−^-N, TP, TN and other aquatic environmental factors. Lu et al. [18] observed that sediment microbial community abundance was significantly influenced by temperature. Generally, the determining factors of microbial communities in different ecosystems varied.

Sediment microbial community structure and abundance determines the main biological processes and efficiency of biogeochemical cycles, e.g., ammonia oxidation and nitrification [19]. NH_4_^+^-N is the original substrate of the ammonia oxidation process [20]. Some products of the ammonia oxidation provide the original substrate for the nitrification process, such as NO_3_-N. Thus, the distribution of dominant sediment microbial taxa generally varies with the concentration gradients of NH_4_^+^-N [21]. Ammonia-oxidizing bacteria (AOB) are the driving microorganisms of the ammonia oxidation process and ammonia-oxidizing archaea (AOA) also influence this process. Zhang et al. [22] found that AOA abundance was limited by NO_3_^−^N as a result of substrate inhibition effects, but its diversity would increase as a result of increasing ecological niche width. There also exist controversial conclusions on the effects of TOC on AOA. Some AOA taxa were positively correlated with TOC since TOC was the carbon source of their growth [23]. *Nitrososphaera viennensis* generally had high growth rate in the presence of pyruvate. However, the growth of some other AOA taxa, such as *Candidatus nitrosotaleade vanaterra*, was limited by TOC [24]. Therefore, the effects of TOC on AOA are determined by the structure of microbial community. The affinity of AOA in using NH_4_^+^-N is much higher than that of AOB, but its use efficiency is relatively low under acid conditions. As a result, AOA often dominates in alkaline states with a wide range of NH_4_^+^-N concentrations, while AOB dominates in relatively high concentrations of NH_4_^+^-N [25]. AOB are typical inorganic autotrophic microorganisms and are independent of sediment organic carbon. However, Zhang [26] observed that sediment organic carbon benefited the growth of heterotrophic bacteria that prevented the growth of AOB through exploitative competition, leading to a negative correlation between AOB and organic carbon.

The structure of the sediment microbial community also influences the circulation of phosphorus [27]. Various forms of phosphorus exist in sediments, including NaOH-P in the form of oxides and hydroxides, HCl-P combined with Ca, and organic phosphorus (OP). The sediment microbial community can release sediment phosphorus into water [28]. The activity of the microbial community and alkaline phosphatase in the sediment are recognized as key factors influencing the interconversion of various phosphorus forms [29]. Lactobacillus can decompose insoluble phosphate and increase the soluble phosphorus concentration in water [30]. Besides nutrients, water and sediment temperature also affect the structure of microbial community through influencing their metabolic rates, activity of enzymes, and so on [31]. AOA can conduct the ammonia oxidation process in temperatures ranging from −1 °C in arctic coastal regions to 97 °C in hot springs [32]. However, the optimum temperature of the AOB nitrification process is 15~25 °C [33], which is also the active temperature of most microorganism taxa. Stres et al. [34] showed that AOA was more influenced by temperature than AOB.

In deep lakes or rivers, sediments generally have a relatively stable environment. The temperature of surface sediments in deep waters varies only slightly, even if the air environment fluctuates fiercely. However, in a shallow river, sediment environmental variables may change greatly with the air environment and water flow. How the sediment microbial community structure changes with the fluctuating environmental variables of both water and sediment has been seldom explored. Furthermore, whether the microbial community structure would change significantly from source to downstream has also been rarely analyzed.

In the present study, the main environmental variables and the surface sediment microbial community were measured bimonthly from August 2018 to June 2020 in River Taizicheng, a shallow temperate mountain river at the core area of the 2022 Winter Olympics. The temporal and spatial patterns of environmental factors and the community structure of the sediment microbial community were analyzed. The structural equation model (SEM) was used to explore the pathways by which environmental factors from the water and sediment influenced community abundance and diversity. The effects of water and sediment environmental factors on the sediment microbial community were analyzed using canonical correspondence analysis (CCA). We assumed that the sediment microbial community had high spatial and temporal autocorrelations in this small mountain river. We also assumed that the fluctuations in community structure were strongly affected by environmental variables.

## 2. Materials and Methods

### 2.1. Study Area

**Figure 1:**
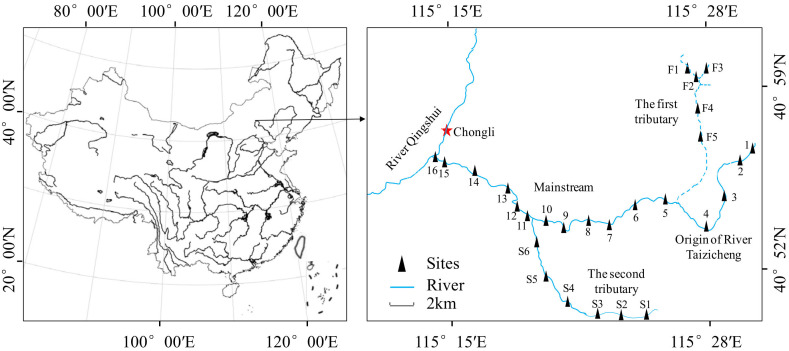
Location of Taizicheng river and the sampling sites.

Taizicheng is a mountain river located at the core area of the 2022 Winter Olympics in Chongli District, Zhangjiakou City, China (Figure 1). It originates from a few mountains covered by forest and flows into the River Qingshui. The river has a length of 27.0 km, and its average width is about 2.0 m. The river is shallow, and the depth of most parts is lower than 0.5 m. The climate of the river area is temperate with an annual average temperature of 3.0 °C and rainfall of about 500 mm. The sediment type of the river is clastic sediment.

### 2.2. Sampling and Measurements

#### 2.2.1. Sampling Methods

A total of 27 sampling sites were evenly set throughout the river (Figure 1). At the origin of the river, we set Sites 1~4. At the first tributary of the river, we set Sites F1~F5. At the second tributary of the river, we set Sites S1~S6. At the mainstream of the river, we set Sites 5~16. All investigations and measurements were conducted bimonthly from August 2018 to June 2020. A few sites were covered by ice or influenced by engineering construction, thus sampling at these sites was paused. Sampling was also paused in February 2020 due to the COVID-19 pandemic. As a result, there were 127 samples collected and measured throughout the river, including 12 samples in August, 8 samples in October, 7 samples in December 2018, 8 samples in February, 12 samples in April, 11 samples in June, 15 samples in August, 15 samples in October, 5 samples in December 2019, 17 samples in April, and 17 samples in June 2020. GPS was used to determine the positions of each site. All samplings and measurements were carried out using the same methods.

#### 2.2.2. Environmental Conditions’ Measurements

Water temperature (WT, °C), pH, DO (mg/L) and electrical conductivity (EC, uS/cm) were measured in situ using YSI Professional Plus. The values of turbidity (Tur) were measured using a portable turbidimeter. Water samples (1 L) were collected using a Tygon tube water sampler. They were stored in acid-cleaned glass bottles at 4 °C and analyzed within 24 h. Two sediment samples were collected at the surface of the sediment using a Peterson dredger at each site, one for biological analysis stored in a 50 mL sterilized plastic jar and the other one for physical and chemical analysis stored in a sterilized Ziploc bag. Water and sediment TN (mg/L), NH_4_^+^-N (mg/L) and TP (mg/L) were measured using potassium persulfate oxidation–UV spectrophotometry method, Nessler’s reagent spectrophotometry method, and Mo-Sb anti-spectrophotometry method [35]. Sediment NaOH-P was measured with the Mo-Sb colorimetric method and HCl-P was measured using the perchloric acid decomposition method.

#### 2.2.3. Metagenomic Analysis

Sediment microbial communities of the 127 sites were measured with the following processes: preparing the samples, DNA extraction and detection, PCR amplification, product purification, gene library construction and testing, and sequence analysis. Microbial genomic DNA was extracted within 24 h using DNeasy^®^ PowerSoil^®^ Kit and stored at −20 °C using the sediment samples. The quality and amount of DNA were checked by agarose gel electrophoresis. Then, 16S rRNA genes of distinct regions (16S V3 + V4/16S V4 + V5) were amplified using the specific primer (341F: CCTAYGGGRBGCASCAG, 806R: GGACTACNNGGGTATCTAAT, Arch519F: CAGCCGCCGCGGTAA, Arch915R: GTGCTCCCCCGCCAATTCCT) with a barcode. Phusion^®^ High-Fidelity PCR Master Mix with GC Buffer and high-fidelity enzymes were used to conduct PCR. PCR products were detected by the gel electrophoresis method (with 2% agarose gel). PCR products were mixed in equidensity ratios. Then, mixed PCR products were purified with Qiagen Gel Extraction Kit (Qiagen, Stockach, Germany). Sequencing libraries were generated using TruSeq^®^ DNA PCR-Free Sample Preparation Kit (Illumina, San Diego, CA, USA) following manufacturer’s recommendations, and index codes were added. The library quality was assessed on the Qubit@ 2.0 Fluorometer (Thermo Fisher Scientific, Waltham, MA, USA) and Agilent Bioanalyzer 2100 system. The library was sequenced on an Illumina NovaSeq platform and 250 bp paired-end reads were generated. Quality filtering on the raw tags was performed under specific filtering conditions to obtain the high-quality clean tags according to the QIIME (V1.9.1) quality-controlled process. The tags were compared with the reference database (Silva database) using UCHIME algorithm (UCHIME) to detect chimera sequences, and then the chimera sequences were removed. Then, the Effective Tags were finally obtained. Based on the results of comparing 16S rRNA gene sequences, operational taxonomic unit (OTU) clustering (with 97% similarity) and species annotation were carried out after preliminary processing of sequencing data using the Silva Database [36,37,38,39]. Finally, taxonomic information was obtained, and the community composition of each sample was counted at each classification level. The biological detection was conducted at the NoveGene Company at Beijing China.

### 2.3. Statistical Analysis

The number of observed species and Chao1 index were used to show the species richness of microbial community. Shannon–Wiener and Simpson indices were used to reflect the diversity of community. Shannon–Wiener diversity index was calculated with the following equation:

(1) $$H=-\sum_{i=1}^{S}\frac{ni}{N}\ln(\frac{ni}{N})$$

Here, *H* is Shannon–Wiener diversity index, *S* is the number of OTUs, *n_i_* was the number belonging to the *i*th OTUs, and *N* was the total number.

Microbial community Simpson diversity index was calculated with the following equation:

(2) $$D=1-\frac{\sum_{i=1}^{S}ni(ni-1)}{N(N-1)}$$

Here, *D* is Simpson diversity index. The variables *S*, *n_i_* and *N* were the same as those in Equation (1). Microbial community Chao1 index was calculated with the following equation:

(3) $$\mathrm{Chao}1=S+\frac{F_{1}{}^{2}}{2F_{2}},\;\mathrm{Chao}1=S+\frac{F_{1}(F_{1}-1)}{2(F_{2}+1)}$$

Here, *S* is the total number of OTUs, *F*_1_ means the number of OTUs when there was only one read and *F*_2_ means the number of OTUs when there were only two reads. The former equation was used when *F*_2_ was nonzero and the latter one was used when *F*_2_ was zero.

The significant differences in microbial community diversity in different months were compared using Analysis of Variance (ANOVA). The differences in environmental factors across months were also analyzed using ANOVA. Before analysis, Kolmogorov–Smirnov was used to test the normally distribution of the data and Bartlett test was used to calculate the homogeneity of data variance. Post hoc comparisons were applied using the Tukey HSD test at a significance level of 0.05. The diversity indices and ANOVA were calculated through SPSS.

The similarity between microbial communities from different sites was calculated by 1-Euclidean distance after min–max normalization, and thus a high value meant high similarity. To show the similarity among microbial communities from different measurements, we conducted detrended correspondence analysis (DCA) using the number of organisms at the genera level. The species matrix included the abundance of dominant microbial community genera whose average abundance proportion was within the top 20. The analysis was calculated using Canoco.

We used SEM to quantify the relationship between environmental variables and the microbial community. The initial model included all environmental factors from both water and sediment since they are considered to have a direct influence on microbial community in this shallow river, on the basis of previous literature. We also specified covariance paths among environmental variables in the initial model. The pathways with high *p*-values were eliminated gradually from the model. The best-fitted model was the one with the lowest values of *χ*^2^-statistic (*p* > 0.05), Akaike information criteria (AIC) and root-mean-square error of approximation (RMSEA). We used SEM to identify the pathways by which the environmental variables in water and sediment influenced microbial community abundance and diversity. The calculation of the model was conducted via SPSS AMOS (Analysis of Moment Structure).

The effects of water and sediment environmental factors on microbial community were analyzed using CCA. In the statistics, the environmental matrix included the values and concentrations of WT, DO, pH, EC, Tur, TN, NH_4_^+^-N and TP. The species matrix included the abundance of dominant microbial community phyla whose average abundance proportion was in top 20. All the variables of environmental and biological factors were transformed by log_10_(*x* + 1) except for pH. CCA was calculated using Canoco for Windows 4.5, and the figures were obtained through Canodraw for Windows.

## 3. Results

### 3.1. Environmental Variables of Water and Sediment in the River

**Figure 2:**
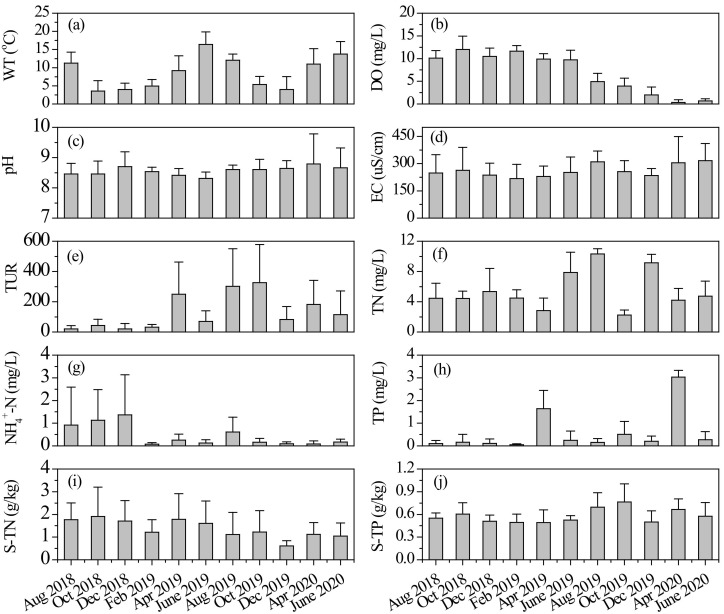
Average values of (**a**) water temperature, (**b**) dissolved oxygen, (**c**) pH, (**d**) electrical conductivity, (**e**) turbidity, (**f**) total nitrogen, (**g**) ammonia nitrogen, (**h**) total phosphorus, (**i**) sediment total nitrogen, and (**j**) sediment total phosphorus in different months, values expressed as mean ± standard deviation. S-TN: sediment total nitrogen, S-TP: sediment total phosphorus.

During our research, both water and sediment environmental factors showed significant variations over time (Figure 2). Average values of WT varied from 3.5 ± 2.9 °C (mean ± standard deviation) to 16.4 ± 3.5 °C (Figure 2). There were significant differences in DO concentration among different measurements (one-way ANOVA: *F* = 96.55, *p* < 0.001). Its average values in the last five investigations were significantly lower than that in other months (all *p* < 0.001, post hoc Tukey HSD). Mean pH and EC values were relatively constant among different samplings (one-way ANOVA, *p* > 0.05), as shown in Figure 2. The values of pH indicated a weak alkaline state of the river. Water Tur fluctuated fiercely (one-way ANOVA: *F* = 5.29, *p* < 0.001) in this mountain river, and its values in some months of 2019 were significantly higher than others. The river had relatively high nutrient concentrations. Average values of water TN ranged from 2.25 ± 0.66 mg/L to 10.32 ± 0.69 mg/L and NH_4_^+^-N from 0.08 ± 0.06 mg/L to 1.37 ± 1.77 mg/L. Different from other nutrients, average NH_4_^+^-N concentrations in the river decreased gradually during the study, as shown in Figure 2. Sediment TN concentration varied between 0.61 ± 0.23 g/kg and 1.91 ± 1.29 g/kg (Figure 2). The average concentration of sediment TP in October 2019 was significantly higher than that in other months (all *p* < 0.05, post hoc Tukey HSD).

### 3.2. Sediment Microbial Community Diversity and the Driving Factors

**Figure 3:**
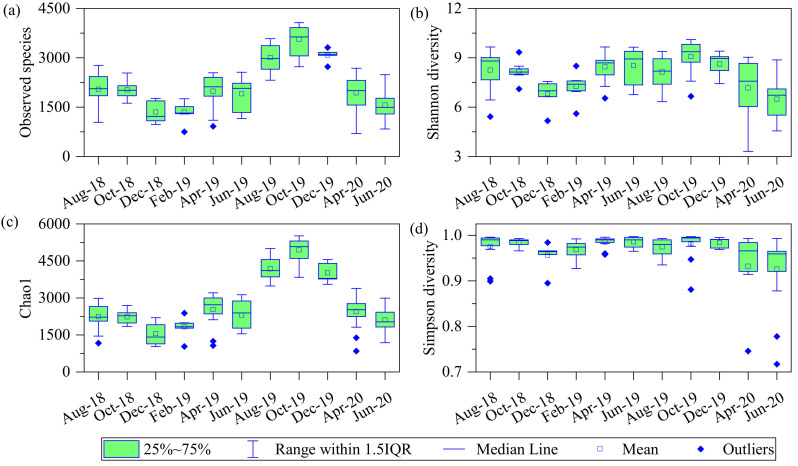
Average values of microbial community (**a**) observed species, (**b**) Shannon–Wiener diversity indices, (**c**) Chao1 diversity indices, and (**d**) Simpson diversity indices in different months.

During the study, 49 phyla, 65 classes, 128 orders, 240 families, 840 genera of bacteria and 5 phyla, 8 classes, 13 orders, 18 families, 45 genera of archaea were identified in the river. The number of observed species showed significant fluctuations across months (one-way ANOVA: *F* = 29.20, *p* < 0.001), with average values in October 2019 higher than that of all other months (*p* < 0.05 for all, post hoc Tukey HSD). Furthermore, the number of observed species was relatively high in summer and low at winter (Figure 3). The average Shannon–Wiener, Chao1 and Simpson indices all reached their highest value in October 2019 and were significantly higher then than in other months (*p* < 0.05 for all, post hoc Tukey HSD). All in all, the diversity of the microbial community showed seasonal fluctuations and reached its highest value in autumn.

**Figure 4:**
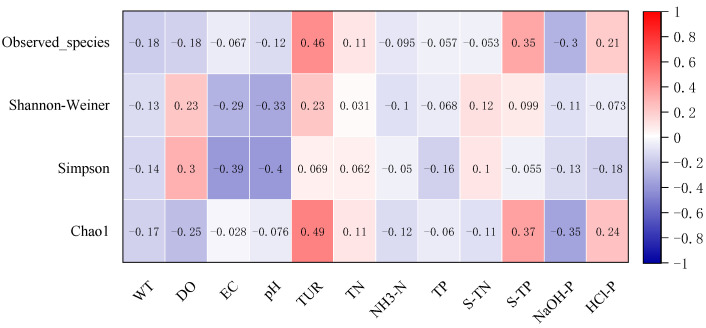
Correlation coefficients among environmental variables and microbial community diversity indices.

The number of observed species and Chao1 indices of the sediment microbial community were significantly positively correlated with Tur (*p* < 0.001) and sediment TP (*p* < 0.001), but had a strong negative relationship with NaOH-P (*p* < 0.001, Figure 4). The number of observed species increased with Tur. Tur also had strong positive relationship with Chao1 index (*r* = 0.49, *p* < 0.001). EC and pH were significantly negatively related to Shannon–Wiener and Simpson diversity indices (*p* < 0.001), while the number of observed species and Chao1 index were negatively correlated with NaOH-P (*p* < 0.001).

The best-fitted SEM model to explain the number of microbial observed species included Tur, TP, DO, EC and sediment TN (Table 1). The standardized partial regression coefficient of Tur in the model was 0.59, indicating a strong positive influence on the observed species. The model’s *χ*^2^ value was 0.021 (*p* = 0.884) and its RMSEA was lower than 0.001, indicating a significant influence of the environment on diversity index (Table 2). The best-fitted SEM model to explain the Shannon–Wiener diversity index included pH, Tur and EC (*χ*^2^ = 0.522, *p* = 0.470, Table 1). The standardized partial regression coefficients of the three variables were −0.28, 0.31 and −0.36, respectively. Four environmental variables, pH, Tur, EC and TP, were selected in the best-fitted SEM to explain Simpson diversity index (*χ*^2^ = 1.492, *p* = 0.474, Table 1), and their standardized partial regression coefficients were −0.34, 0.20, −0.40 and −0.14, respectively. The factors that had direct influence on Chao1 index were similar to those of the observed species. All in all, Tur and sediment nutrients generally had a positive influence and water nutrients had a negative influence on microbial community diversity.

### 3.3. Temporal–Spatial Patterns of Sediment Microbial Community

**Figure 5:**
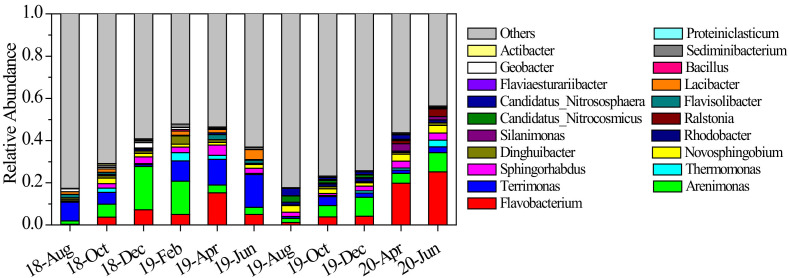
Average abundance proportions of dominant sediment microbial genera from August 2018 to June 2020. Values are expressed as the mean of sampling sites.

**Figure 6:**
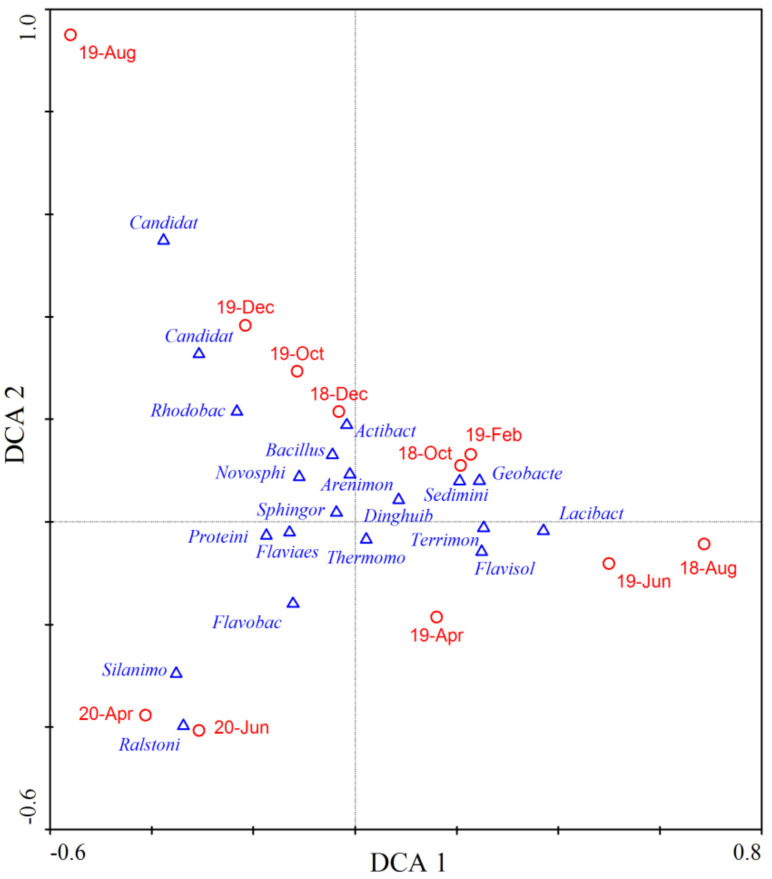
DCA plots showing the average similarity of sediment microbial community between different measurements. Blue triangles stand for dominant genera in the river and red circles for community at different months. The names in the figure are the abbreviation of microbial genera listed in Figure 5.

During our research, Proteobacteria and Bacteroidetes were the most dominant phyla and their average abundance proportion reached 62.5%. From autumn to winter 2018, the abundance proportions of *Flavobacterium* and *Arenimonas* increased, while that of *Terrimonas* decreased (Figure 5). However, the situation reversed from winter 2018 to summer 2019, where *Arenimonas* abundance proportion declined but *Terrimonas* increased. The average abundance proportion of Bacteroidetes continued to increase and it became the most dominant phylum. The microbial community structure varied significantly from summer to autumn 2019, as shown in Figure 6. The abundance proportions of *Flavobacterium*, *Arenimonas* and *Terrimonas* decreased from 23.96% to 3.95% (Figure 5). Archaea genera *Candidatus_Nitrocosmicus* and *Candidatus_Nitrososphaera* increased to 6.39%. From autumn to winter 2019, the abundance proportions of *Flavobacterium* and *Arenimonas* continued to increase. From winter 2019 to summer 2020, the dominance of *Flavobacterium* became enhanced, and its average abundance proportion reached 25.18%. *Novosphingobium* also reached its maximum value in summer 2020 (Figure 5). The average similarity of the microbial community between two adjacent measurements was relatively lower than others in most cases (Figure 6), indicating a high temporal autocorrelation of community structure.

**Figure 7:**
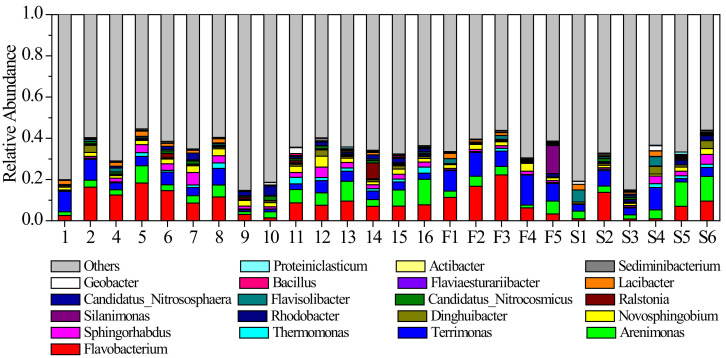
Average abundance proportions of dominant sediment microbial genera at different sites in the river. Values are expressed as the mean of each measurement.

**Figure 8:**
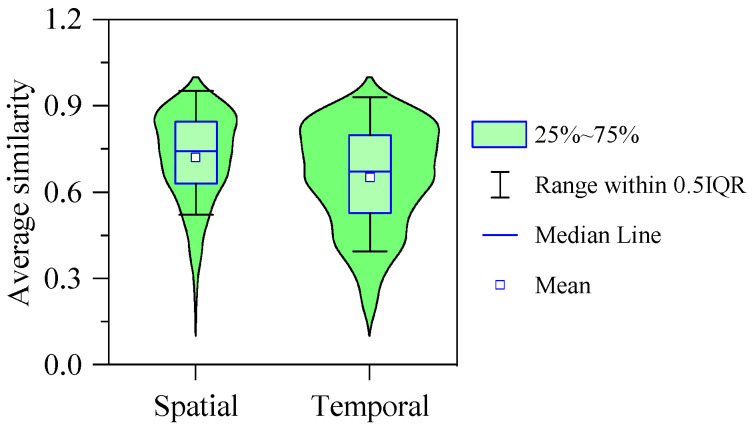
Violin plots showing the average similarity of community structure between sites of spatial and temporal variations.

There were also apparent spatial variations in the sediment microbial community patterns of the river, as shown in Figure 7. The microbial community at the origin of the river was dominated by *Flavobacterium* and *Terrimonas* (Figure 7). These two genera accounted for 18.32% of the total abundance. At the first tributary of the river, the abundance proportion of *Flavobacterium* reached 11.98% and was significantly higher than in other parts. Furthermore, the average proportion of *Terrimonas* was 10.40%. At the second tributary of the river, both *Flavobacterium* and *Terrimonas* were much lower than that at the first tributary (Figure 7). However, the abundance proportion of *Arenimonas* was higher than in other parts (Figure 7). At the mainstream of the river, *Flavobacterium*, *Arenimonas* and *Terrimonas* were also the dominant genera, with average abundance proportions of 8.75%, 5.89% and 4.11%, respectively (Figure 7). The abundance proportion of *Flavobacterium* remained stable in the downstream section of the river (Sites 11 to 16). In addition, the spatial similarity of the community structure was significantly higher than temporal similarity (Figure 8).

**Figure 9:**
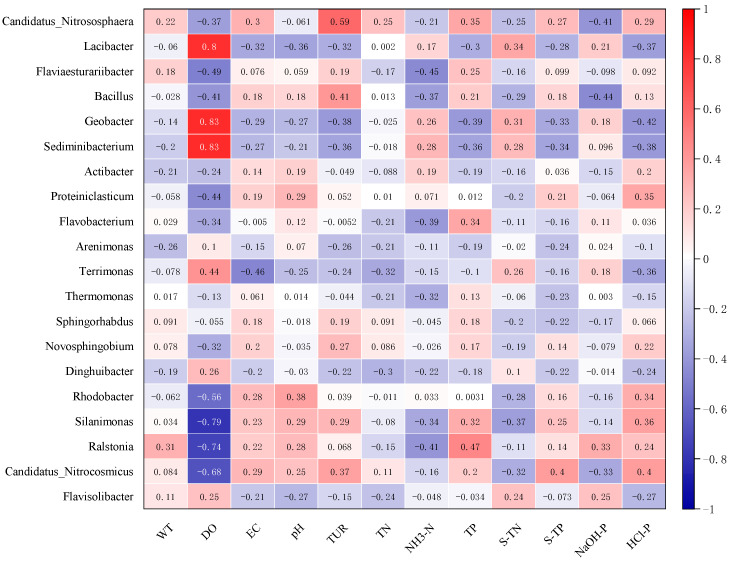
Correlation coefficients between environmental factors and dominant microbial genera abundance.

Figure 9 indicates that *Flavobacterium* had strong positive correlation coefficients with TP (*p* < 0.001) but was negatively correlated with DO (*p* < 0.001). The abundance of *Terrimonas* was significantly positively related to DO (*p* < 0.001) and S-TN (*p* < 0.01), while its correlations with EC, HCL-P, TN, pH and Tur were negative. The abundance of *Arenimonas* was negatively influenced by most environmental variables (Figure 9). The correlation between environmental factors and the abundance of the microbial community was complex. Many variables had contrary relationships with different microbial genera.

The best-fitted SEM model to explain the abundance of Bacteroidetes included EC, TN, WT, DO and Chao1 index (*χ*^2^ = 0.618, *p* = 0.734, Table 1). We added the four diversity indices in the initial model since biodiversity would influence abundance in many biological communities. These environmental factors influenced the abundance of Bacteroidetes directly, with standardized partial regression coefficients of −0.23, 0.23, 0.25 and 0.15, respectively. On the other hand, TN, WT and DO also affected Bacteroidetes indirectly through influencing its diversity, with standardized partial regression coefficients of 0.22, −0.34 and −0.35. Similar patterns were found in the best-fitted SEM model to explain the abundance of Proteobacteria (Table 1). WT, EC, Tur and sediment TP were chosen in the model, with standardized partial regression coefficients of −0.29, 0.36, −0.13 and −0.10, respectively. HCl-P did not have direct influence on the abundance of Proteobacteria, but it affected Proteobacteria through influencing its diversity index (Table 1). EC, Tur and sediment TP also indirectly affected Proteobacteria through influencing its diversity, with standardized partial regression coefficients of −0.36, 0.37 and 0.23, respectively. In both models, the effects of diversity index on the abundance of the two phyla were negative (Table 1).

### 3.4. Effects of Environmental Factors on Microbial Community

To explore the environmental variables driving the microbial community, we conducted CCA among them. Microbial phyla that were chosen for CCA are listed in Table 3. The environmental axes in the CCA were perpendicular and the species axes 1 and 2 were nearly vertical to each other. Species axis 1 was positively correlated with sediment HCl-P (*R* = 0.40), TP (*R* = 0.39) and water Tur, but negatively correlated with water DO (*R* = −0.85) and sediment TN (*R* = −0.33). Species axis 2 was negatively correlated with WT (*R* = −0.44) and water TP (*R* = −0.32). Monte Carlo testing showed that the first canonical axis and all canonical axes were both significant (*p* = 0.002 for both). The eigenvalues values of axes 1 and 2 were 0.122 and 0.007, respectively. The correlation coefficients between the first two species axes and environmental factors were 0.931 and 0.743, indicating a close relationship between microbial community and the environmental variables. These axes explained 71.6% of the total variance of microbial community.

**Figure 10:**
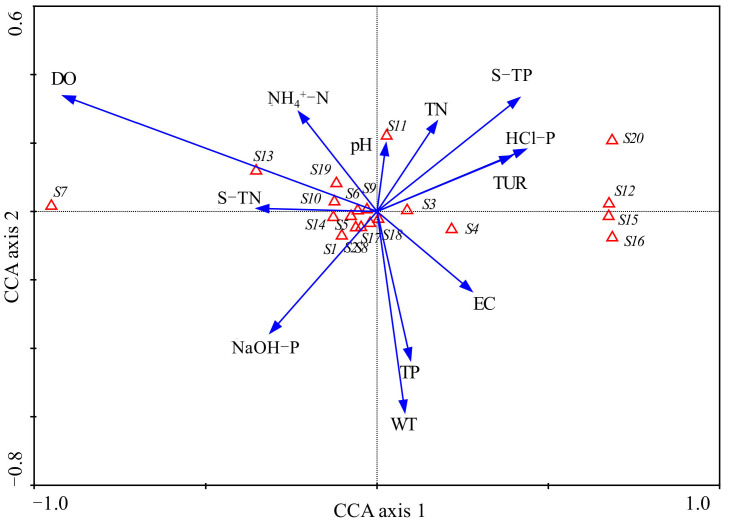
Results of CCA plotted for axis 1 and axis 2. Sediment microbial phyla are represented by an open triangle and the explanations for the symbols used are given in Table 3. Environmental factors are expressed as blue lines with arrows. S-TN: sediment total nitrogen, S-TP: sediment total phosphorus.

The main water and sediment environmental variables affected sediment microbial community distribution to different degrees (Figure 10). Sediment microbial phyla Crenarchaeota, Desulfobacterota, Myxococcota and Methylomirabilota were distributed at the positive direction of the first axis. Their distribution was positively influenced by sediment HCl-P, TP, water Tur and EC, but negatively affected by DO. Water DO was the most significant environment variable that influenced the abundance of Chloroflexi, Actinobacteria, Firmicutes, Planctomycetes, Euryarchaeota and Gemmatimonadetes. In addition, these phyla were positively affected by sediment TN. Archaea phylum Thaumarchaeota was highly positively affected by sediment TN, as shown in Figure 10. The values of pH had a strong positive relationship with the abundance of Cyanobacteria.

**Figure 11:**
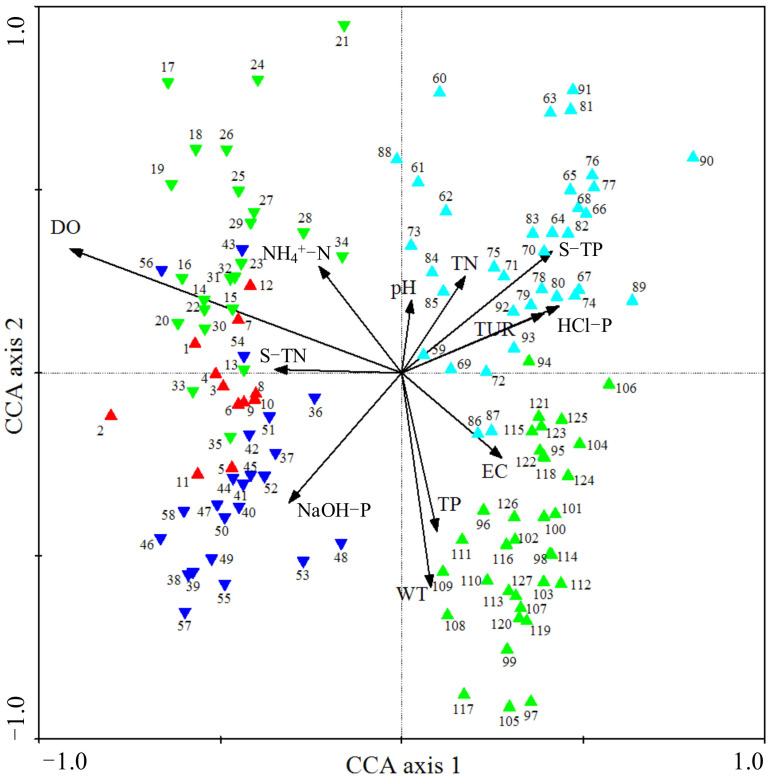
Results of CCA plotted for the effects of main environmental factors on the sediment microbial communities at sites. Environmental factors are expressed as black lines with arrows. S-TN: sediment total nitrogen, S-TP: sediment total phosphorus. Samples from August 2018: red up-triangle; samples from October 2018 to February 2019: green down-triangle; samples from April to June 2019: blue down-triangle; samples from August to December 2019: cyan up-triangle; samples from April to June 2020: green up-triangle.

Samples in August 2018 (1~12, red up-triangle in Figure 11) were distributed in the negative direction of the first axis, and they were mainly influenced by water DO and sediment TN. Sediment microbial community samples from October 2018 to February 2019 were distributed in the third quadrant (13~35, green down-triangle in Figure 11), and they were mainly affected by water DO and NH_4_^+^-N. Sediment NaOH-P was the main factor that determined the distribution of samples in late spring and summer 2019 (36~58, blue down-triangle in Figure 11). However, from August to December 2019, the sediment microbial community at different sites (59~93, cyan up-triangle in Figure 11) were mainly affected by the concentrations of sediment TP, HCl-P, water Tur and TN. The values of WT, TP and EC were the main factors that determined the sediment microbial community in April and June 2020 (94~127, green up-triangle in Figure 11). Sites within the same months had low distance and high similarity, indicating a high spatial autocorrelation of microbial community structure.

## 4. Discussion

The interaction between biological and environmental factors has long been a hot topic in ecology. Owing to their small body masses, sediment microbial communities are sensitive to the fluctuations of environment. In this study, we observed the temporal and spatial patterns of sediment microbial community structure based on field investigations in a shallow mountain river. The microbial community had high spatial and temporal autocorrelation. The pathways by which environmental factors from water and sediment influenced microbial community abundance and diversity were explored. The driving environmental factors of these variations were shown. Our results helped us to understand the microbial community patterns and their driving factors.

### 4.1. Microbial Community Structure

During the study, Proteobacteria and Bacteroidetes were the most dominant microbial phyla, with average abundance proportions of 32.26% and 30.24%, respectively. Proteobacteria has high levels of species and genetic diversity and covers a wide range of physiological metabolic types, including aerobic, anaerobic, autotrophic and heterotrophic bacteria. These properties mean that Proteobacteria is widely distributed throughout natural ecosystems [40]. Newberry et al. [41] found that Proteobacteria was the dominant phyla using phylogenetic analysis based on PCR amplification and sequence analysis of 16S rRNA and methyl co-enzyme M reductase genes in deep marine sediments. Li et al. [42] showed that Proteobacteria occupied 76% of the clone library in surface sediments from deep waters west of Japan. Marchesi et al. [43] analyzed approximately 400 bacterial clones using phylogenetic analysis and found that 96% were members of the Proteobacteria in deep sea sediment. Martins et al. [44] observed that the dominant members of sediment bacterial community were mostly affiliated with the Proteobacteria phylum in eutrophic Azorean lakes. Lin et al. [45] also suggested that Proteobacteria was the most dominant phyla, and its abundance proportion varied from 45.3% to 69.1% in different sites in the Guishui river. Zhao et al. [46] showed that the abundance proportion of Proteobacteria ranged between 25.1% and 49.4% at different sites in a typical plateau lakeshore. Combining these results, we can conclude that Proteobacteria is the dominant phylum in both freshwater and deep-sea ecosystems. However, its abundance proportion in freshwater ecosystems [45,46] is much lower than that in deep-sea sediments [42,43]. Bacteroidetes can be found in just about every habitat in the biosphere, and it can be the main phylum even in saline alkali environments [47,48,49]. Kirchman [47] found that the entirety of Bacteroidetes represented about 5% of all 16S rRNA sequences. Zhong et al. [50] showed that the average abundance proportion of Bacteroidetes accounted for 11% of total sediment microbial community abundance. The average abundance of Bacteroidetes in this research was much higher than that in other studies.

We observed that *Flavobacterium* was the most dominant genus in the river. *Flavobacterium* facilitates the removal of phosphorus and organic matter [51]. River Taizicheng has high concentrations of TP (Figure 2) and organic matter since it originates from a few mountains covered by forest. Furthermore, the growth and death of Cyanobacteria in the river also increase the abundance of *Flavobacterium*. *Arenimonas* and *Terrimonas* are generally associated with denitrification, which reduces nitrate and removes total nitrogen from the river [52,53,54]. Therefore, high concentrations of TN and TP determined the structure of the microbial community.

### 4.2. The Relationship between Microbial Community and Environment

We observed the pathways by which environmental factors from water and sediment influenced microbial community abundance, which helped to explain their temporal fluctuations. Water temperature, TN and DO promoted the abundance of Bacteroidetes, while the influence of EC was negative. Proteobacteria were positively affected by EC but negatively influenced by water temperature. From autumn to winter 2018, the abundance proportion of Bacteroidetes decreased while that of Proteobacteria increased. Water temperature was the main driving variable of this change. From February to June 2019, Bacteroidetes abundance increased while that of Proteobacteria decreased. Water temperature and EC played the biggest role in these changes. From February to June 2020, the abundance of Bacteroidetes increased gradually and water temperature was the main influencing factor.

Environmental variables not only affected microbial community abundance, they also influenced the diversity of the community. We observed that turbidity had positive effects on sediment microbial community diversity index. Generally, turbidity has negative effects on aquatic biology since solar radiation decreases with turbidity. The benefit effects of Tur indicated that the sediment microbial community was suited to a dark environment. Furthermore, we also observed that sediment nutrients generally had a positive influence while water nutrients had a negative influence on microbial community diversity. Gilbert et al. [55,56] showed the seasonal structure of microbial communities in the western English Channel and found that high richness and diversity indices of microbial communities were found in winter, when nutrients were enriched.

SEM showed that environmental variables not only affected sediment microbial community abundance directly, they also had an influence on them indirectly through influencing their diversity. The relationship between biodiversity and ecosystem function has fascinated ecologists for decades. There have been experimental studies that have shown positive, negative, neutral, or even more complex patterns between biodiversity and community productivity in aquatic ecosystems [57,58,59]. However, the relationship between sediment microbial community diversity and ecosystem function has been seldom explored. Our results indicated that Shannon–Wiener and Chao1 diversity both had a negative influence on sediment microbial community abundance.

The sediment microbial community had a high spatial autocorrelation in the river. The community structure upstream would have influence on that at downstream due to river fluidity, especially in this mountain river. The high similarity of the microbial community at adjacent sites confirmed this conclusion (Figure 11). However, there were also apparent spatial variations in microbial community structure. At the origin of the river, the abundances of Bacteroidetes and Crenarchaeota were relatively high. At the mainstream of the river, Proteobacteria abundance proportion reached its highest value. At the first tributary of the river, Bacteroidetes was the most dominant phylum. Combining the fluctuations in environmental variables with CCA and SEM, we could conclude that DO, EC and nutrients were the main variables driving these changes.

CCA showed that DO, WT and various forms of phosphorus were the main factors affecting the distribution of sediment microbial communities. Phosphorus is the original material of biochemical reactions of microbial communities. Temperature and DO affected the growth of sediment microorganism through influencing metabolic rates, activity of enzymes, and so on [31]. Lin et al. [45] showed that water ammonia nitrogen and temperature were the main environmental factors influencing the microbial communities and that Bacteroidales was mostly correlated with ammonia nitrogen using redundancy analysis on the sediment of River Guishui. Zhao et al. [46] suggested that TP was the main environmental factor that influenced the distribution of the sediment microbial community in a typical plateau lakeshore. Lu et al. [18] observed that sediment microorganism community abundance was significantly influenced by temperature. Hou [15] studied the sediment microbial community distribution and found that there was a negative relationship between microorganism community abundance and the concentration of organic matter in the sediments of Lake Guozheng and Lake Tuanhu.

The microbial community links biotic and abiotic resources in natural ecosystems. The sediment microbial community structure affects the material circulation and energy flow of aquatic ecosystems. In this research, we used SEM to explore the pathways by which environmental factors from water and sediment influenced microbial community abundance and diversity. The relationship between environmental variables and microbial community were standardized by partial regression coefficients. This helped us to obtain the magnitude and direction of the effects of an independent variable on the sediment microbial community and eliminated the impact of other variables. In addition, we used a two-year investigation to conduct our statistics, which increased the accuracy and reliability of our results. In the future, long-term and high-frequency monitoring will be critical in exploring the driving factors of sediment microbial communities in complex natural ecosystems.

## 5. Conclusions

Based on field investigations and high-throughput 16S rRNA genes sequencing technologies, the temporal and spatial patterns of environmental variables and sediment microbial community structure were explored in River Taizicheng, a temperate monsoon shallow mountain river in the core area of the 2022 Winter Olympics. The pathways by which environmental factors from water and sediment influenced microbial abundance and diversity were investigated through SEM. The effects of environmental factors on the temporal and spatial fluctuations of the microbial community were analyzed based on CCA. As a result, the following can be concluded.

(1) The observed species, Shannon–Wiener, Chao1 and Simpson diversity indices of the microbial community were positively influenced by turbidity and sediment nutrients but negatively affected by water nutrients.

(2) Bacteroidetes and Proteobacteria were the most dominant phyla during the research. The best-fitted SEM model indicated that environmental variables not only affected microbial community abundance directly, but also indirectly through influencing their diversity.

(3) *Flavobacterium*, *Arenimonas* and *Terrimonas* were the dominant genera as a result of enriched nutrient concentration. They also facilitated the removal of phosphorus and nitrogen from the river.

(4) The microbial community had high spatial–temporal autocorrelations in this small, shallow, temperate mountain river.

(5) CCA showed that DO, WT and various forms of phosphorus were the main variables affecting the temporal and spatial patterns of microbial community abundance in the river.

The results of this research will be helpful in understanding the temporal and spatial patterns of the microbial community and their influencing factors in a mountain river in a temperate monsoon area.

## Figures and Tables

**Table 1:** The best-fitted structural equation models to determine the influence of environmental variables on sediment microbial community diversity index, Bacteroidetes and Proteobacteria abundance. Values are expressed as the standardized partial regression coefficients that were significant in the model.

Explained Variables	Observed Species	Shannon–Wiener	Simpson Diversity	Chao1 Diversity	Bacteroidetes Abundance	Proteobacteria Abundance
WT					0.25	−0.29
DO	−0.30			−0.29	0.15	
EC	−0.27	−0.36	−0.40	−0.28	−0.23	0.36
pH		−0.28	−0.34			
Tur	0.59	0.31	0.20	0.55		−0.13
TN					0.23	
TP	−0.32		−0.14	−0.33		
S-TN	0.12					
S-TP				0.24		−0.10

**Table 2:** The goodness of the best-fitted SEM models to explain the diversity and abundance of microbial community. Models were selected based on significant *χ*^2^ values (*p* > 0.05) and lowest AIC value.

Explained Variables	DF	*χ*^2^ Values	*p*	AIC	RMSEA
Observed species	1	0.021	0.884	52.021	<0.001
Shannon–Wiener	1	0.522	0.470	26.522	<0.001
Simpson Diversity	2	1.492	0.474	37.492	<0.001
Chao1 index Diversity	1	1.319	0.251	53.319	0.050
Bacteroidetes abundance	2	0.618	0.734	50.618	<0.001
Proteobacteria abundance	5	8.188	0.146	68.188	0.050

**Table 3:** Symbols for microbial phyla used in canonical correspondence analysis.

Symbol	Scientific Name	Symbol	Scientific Name
S1	Bacteroidetes	S2	Proteobacteria
S3	Crenarchaeota	S4	Unidentified_Bacteria
S5	Chloroflexi	S6	Actinobacteria
S7	Thaumarchaeota	S8	Acidobacteria
S9	Firmicutes	S10	Planctomycetes
S11	Cyanobacteria	S12	Desulfobacterota
S13	Euryarchaeota	S14	Gemmatimonadetes
S15	Myxococcota	S16	Methylomirabilota
S17	Nitrospirae	S18	Latescibacteria
S19	Verrucomicrobia	S20	Nanoarchaeota

## Data Availability

The data that support the findings of this study are available from the corresponding author upon reasonable request.

## References

[1] Wang X., Wang C., Bao L., Xie S. (2014). Abundance and community structure of ammonia-oxidizing microorganisms in reservoir sediment and adjacent soils. Appl. Microbiol. Biotechnol..

[2] Wagner-Döbler I., Pipke R., Timmis K.N., Dwyer D.F. (1992). Evaluation of aquatic sediment microcosms and their use in assessing possible effects of introduced microorganisms on ecosystem parameters. Appl. Environ. Microbiol..

[3] Weber K., Urrutia M.M., Churchill P.F., Kukkadapu R.K., Roden E.E. (2006). Anaerobic redox cycling of iron by freshwater sediment microorganisms. Environ. Microbiol..

[4] Wu X., Yang X.E., Rengel Z. (2009). Phytoremediation facilitates removal of nitrogen and phosphorus from eutrophicated water and release from sediment. Environ. Monit. Assess..

[5] Klein R., Tischler J.S., Mühling M., Schlömann M. (2013). Bioremediation of Mine Water. Tools Appl. Biochem. Eng. Sci..

[6] Wang F., Dong W., Zhao Z., Wang H., Li W., Chen G., Wang F., Zhao Y., Huang J., Zhou T. (2021). Heavy metal pollution in urban river sediment of different urban functional areas and its influence on microbial community structure. Sci. Total Environ..

[7] Zhang L., Wang S., Li Y., Zhao H., Qian W. (2015). Spatial and temporal distributions of microorganisms and their role in the evolution of Erhai Lake eutrophication. Environ. Earth Sci..

[8] Korlević M., Ristova P.P., Garić R., Amann R., Orlić S. (2015). Bacterial Diversity in the South Adriatic Sea during a Strong, Deep Winter Convection Year. Appl. Environ. Microbiol..

[9] Shi M., Wen G., Liu H., Jian G., Chen Y. (2019). Influence of initial pH on bioleaching of river sediments to achieve deep dehydration. Environ. Sci. Pollut. Res..

[10] Chang W., Sun J., Pang Y., Zhang S., Xu R. (2020). Effects of different habitats on the bacterial community composition in the water and sediments of Lake Taihu, China. Environ. Sci. Pollut. R..

[11] Hunter E.M., Mills H.J., Kostka J.E. (2006). Microbial Community Diversity Associated with Carbon and Nitrogen Cycling in Permeable Shelf Sediments. Appl. Environ. Microbiol..

[12] Gupta R.S. (2013). Molecular Markers for Photosynthetic Bacteria and Insights into the Origin and Spread of Photosynthesis. Adv. Bot. Res..

[13] Hook S.E., Wright A.-D.G., McBride B.W. (2010). Methanogens: Methane Producers of the Rumen and Mitigation Strategies. Archaea.

[14] Levy-Booth D.J., Prescott C.E., Grayston S.J. (2014). Microbial functional genes involved in nitrogen fixation, nitrification and denitrification in forest ecosystems. Soil Biol. Biochem..

[15] Hou J., Cao X., Song C., Zhou Y. (2013). Predominance of ammonia-oxidizing archaea andnirK-gene-bearing denitrifiers among ammonia-oxidizing and denitrifying populations in sediments of a large urban eutrophic lake (Lake Donghu). Can. J. Microbiol..

[16] Zhao D.-Y., Luo J., Zeng J., Wang M., Yan W.-M., Huang R., Wu Q.L. (2013). Effects of submerged macrophytes on the abundance and community composition of ammonia-oxidizing prokaryotes in a eutrophic lake. Environ. Sci. Pollut. Res..

[17] Liu B., Li Y., Zhang J., Zhou X., Wu C. (2014). Abundance and Diversity of Ammonia-Oxidizing Microorganisms in the Sediments of Jinshan Lake. Curr. Microbiol..

[18] Lu S., Liao M., Xie C., He X., Li D., He L., Chen J. (2015). Seasonal dynamics of ammonia-oxidizing microorganisms in freshwater aquaculture ponds. Ann. Microbiol..

[19] Wei H., Lin X. (2021). Shifts in the relative abundance and potential rates of sediment ammonia-oxidizing archaea and bacteria along environmental gradients of an urban river–estuary–adjacent sea continuum. Sci. Total Environ..

[20] Long Y., Jiang X., Guo Q., Li B., Xie S. (2017). Sediment nitrite-dependent methane-oxidizing microorganisms temporally and spatially shift in the Dongjiang River. Appl. Microbiol. Biotechnol..

[21] Karner M.B., Delong E.F., Karl D. (2001). Archaeal dominance in the mesopelagic zone of the Pacific Ocean. Nature.

[22] Zhang C.L., Ye Q., Huang Z., Li W., Chen J., Song Z., Zhao W., Bagwell C., Inskeep W.P., Ross C. (2008). Global Occurrence of Archaeal amoA Genes in Terrestrial Hot Springs. Appl. Environ. Microbiol..

[23] Lehtovirta-Morley L.E., Stoecker K., Vilcinskas A., Prosser J.I., Nicol G.W. (2011). Cultivation of an obligate acidophilic ammonia oxidizer from a nitrifying acid soil. Proc. Natl. Acad. Sci. USA.

[24] Martens-Habbena W., Qin W., Horak R.E., Urakawa H., Schauer A.J., Moffett J.W., Armbrust E.V., Ingalls A.E., Devol A.H., Stahl D.A. (2014). The production of nitric oxide by marine ammonia-oxidizing archaea and inhibition of archaeal ammonia axidation by a nitric oxide scavenger. Environ. Microbiol..

[25] Boer W.D., Kowalchuk G.A. (2001). Nitrification in Acid Soils: Micro-Organisms and Mechanisms. Soil Bio. Biochem..

[26] Zhang W. (2002). Enrichment and Culture of Nitrifying Bacteria and PCR Amplification of Ammonia Monooxygenase Gene Fragment.

[27] Campos M., Rilling J.I., Acuña J.J., Valenzuela T., Larama G., Peña-Cortés F., Ogram A., Jaisi D.P., Jorquera M.A. (2021). Spatiotemporal variations and relationships of phosphorus, phosphomonoesterases, and bacterial communities in sediments from two Chilean rivers. Sci. Total Environ..

[28] Li B., Ding S.M., Fan C.X., Zhong J.C., Zhang L., Yin H.B., Zhao B. (2008). Distributions of nitrogen and phosphorus in interstitial waters in the sediments of Fubao Bay in Lake Dianchi and their relationships with the activities of microbe and alkaline phosphatase in the surface sediments. J. Lake Sci..

[29] Xia X.H., Dongye M.X., Zhou J.M., Tian S.P., Zhang Z., Peng Y.H. (2002). Geochemistry and Influence to Environment of Phosphorus in Modern Sediment in Dianchi Lake. Acta Sedimentol. Sin..

[30] Wang Y.C., Wan G.J., Wang S.L., Li S.H., Huang R.G. (2000). Forms of phosphorus in sediments of lake Baihua and Lake Hongfeng, Guizhou. Acta Mineral. Sin..

[31] Reigstad L.J., Richter A., Daims H., Urich T., Schwark L., Schleper C. (2008). Nitrification in terrestrial hot springs of Iceland and Kamchatka. FEMS Microbiol. Ecol..

[32] Kalanetra K.M., Bano N., Hollibaugh J.T. (2009). Ammonia-oxidizing archaea in the Arctic Ocean and Antarctic coastal waters. Environ. Microbial..

[33] Avrahami S., Werner L., Ralf C. (2003). Effects of temperature and fertilizer on activity and community structure of soil ammonia oxidizers. Environ. Microbiol..

[34] Stres B., Stopar D., Mahne I., Hacin J., Resman L., Pal L., Fuka M.M., Leskovec S., Danevčič T., Mandic-Mulec I. (2008). Influence of temperature and soil water content on bacterial, archaeal and denitrifying microbial communities in drained fen grassland soil microcosms. FEMS Microbiol. Ecol..

[35] American Public Health Association (APHA), American Water Works Association (AWWA), Water Environment Federation (WEF) (1995). Standard Methods for the Examination of Water and Wastewater.

[36] Bokulich N.A., Subramanian S., Faith J.J., Gevers D., Gordon J.I., Knight R., Mills D.A., Caporaso J.G. (2013). Quality-filtering vastly improves diversity estimates from Illumina amplicon sequencing. Nat. Methods.

[37] Haas B.J., Gevers D., Earl A.M., Feldgarden M., Ward D.V., Giannoukos G., Ciulla D., Tabbaa D., Highlander S.K., Sodergren E. (2011). Chimeric 16S rRNA sequence formation and detection in Sanger and 454-pyrosequenced PCR amplicons. Genome Res..

[38] Wang Q. (2007). Naive Bayesian classifier for rapid assignment of rRNA sequences into the new bacterial taxonomy. Appl. Environ. Microbiol..

[39] Quast C., Pruesse E., Yilmaz P., Gerken J., Schweer T., Yarza P., Peplies J., Glöckner F.O. (2013). The SILVA ribosomal RNA gene database project: Improved data processing and web-based tools. Nucl. Acids Res..

[40] Liu J., Chen X., Shu H.-Y., Lin X.-R., Zhou Q.-X., Bramryd T., Shu W.-S., Huang L.-N. (2018). Microbial community structure and function in sediments from e-waste contaminated rivers at Guiyu area of China. Environ. Pollut..

[41] Newberry C.J., Webster G., Cragg B.A., Parkes R.J., Weightman A., Fry J.C. (2004). Diversity of prokaryotes and methanogenesis in deep subsurface sediments from the Nankai Trough, Ocean Drilling Program Leg 190. Environ. Microbiol..

[42] Li L., Kato C., Horikoshi K. (1999). Bacterial diversity in deep-sea sediments from different depths. Biodivers. Conserv..

[43] Marchesi J.R., Weightman A.J., Cragg B.A., Parkes R.J., Fry J.C. (2001). Methanogen and bacterial diversity and distribution in deep gas hydrate sediments from the Cascadia Margin as revealed by 16S rRNA molecular analysis. FEMS Microbial. Ecol..

[44] Martins G., Henriques I., Ribeiro D., Correia A., Bodelier P., Cruz J., de Brito A.G., Nogueira R. (2012). Bacterial Diversity and Geochemical Profiles in Sediments from Eutrophic Azorean Lakes. Geomicrobiol. J..

[45] Lin H., Cai Y.Q., Li B., Dong Y.B., Li Y. (2019). Characteristics of microbial community structure in Guishui River sediment in Beijing. Acta Ecol. Sin..

[46] Zhao L.J., Liu Y.G., Wang Y., Zhao R., Ren W., Xu M.Z. (2020). Bacterial community structure and diversity of sediments in a typical plateau lakeshore. Microbiol. China.

[47] Kirchman D.L. (2002). The ecology of Cytophaga–Flavobacteria in aquatic environments. FEMS Microbiol. Ecol..

[48] Zhou P., Ma Y., Zhang W., Xue Y., Ventosa A., Grant W.D. (2003). Bacterial diversity of the Inner Mongolian Baer Soda Lake as revealed by 16S rRNA gene sequence analyses. Extremophiles.

[49] Dong H., Zhang G., Jiang H., Yu B., Chapman L.R., Lucas C.R., Fields M.W. (2006). Microbial Diversity in Sediments of Saline Qinghai Lake, China: Linking Geochemical Controls to Microbial Ecology. Microb. Ecol..

[50] Zhong L.Q., Li B., Wang M.H., Zhang S.Y., Jiang H.C., Chen X.H., Zhu J., Bian W.J. (2020). Analysis of microbial community structure and their environmental impact factors in the sediment of channel catfish ponds. J. Fish. Sci. China.

[51] Tang C.C. (2018). Performance and Mechanism of Algal-Bacteial Symbiosis System Based on Sequencing Bactch Biofilm-Sludge Reactor for Nitrogen and Phosphorus Removal.

[52] Zhang Z., Han Y., Xu C., Han H., Zhong D., Zheng M., Ma W. (2019). Effect of low-intensity direct current electric field on microbial nitrate removal in coal pyrolysis wastewater with low COD to nitrogen ratio. Bioresour. Technol..

[53] Zhao Y., Jiang B., Tang X., Liu S. (2019). Metagenomic insights into functional traits variation and coupling effects on the anammox community during reactor start-up. Sci. Total Environ..

[54] Chen X., Xu P., Yang C., Wang S., Lu Q., Sun X. (2020). Study of enhanced nitrogen removal efficiency and microbial characteristics of an improved two-stage A/O process. Environ. Technol..

[55] Gilbert J.A., Field D., Swift P., Newbold L., Oliver A., Smyth T., Somerfield P.J., Huse S., Joint I. (2010). The seasonal structure of microbial communities in the Western English Channel. Environ. Microbiol..

[56] Gilbert J.A., Steele J.A., Caporaso J.G., Steinbrück L., Reeder J., Temperton B., Huse S., McHardy A.C., Knight R., Joint I. (2012). Defining seasonal marine microbial community dynamics. ISME J..

[57] Gamfeldt L., Hillebrand H., Jonsson P.R. (2005). Species richness changes across two trophic levels simultaneously affect prey and consumer biomass. Ecol. Lett..

[58] Weis J.J., Cardinale B.J., Forshay K.J., Ives A.R. (2007). Effects of species diversity on community biomass production change over the course of succession. Ecology.

[59] Schmidtke A., Gaedke U., Weithoff G. (2010). A mechanistic basis for under-yielding in phytoplankton communities. Ecology.

